# Decline in objective physical activity over a 10-year period in a Japanese elementary school

**DOI:** 10.1186/s40101-015-0078-y

**Published:** 2015-11-06

**Authors:** Aya Itoi, Yosuke Yamada, Satoshi Nakae, Misaka Kimura

**Affiliations:** Department of Health, Sports and Nutrition, Faculty of Health and Welfare, Kobe Women’s University, 4-7-2 Minatojima Nakamachi, Chuo-ku, Kobe, 650-0046 Japan; Department of Nutritional Science, National Institute of Health and Nutrition, 1-23-1 Toyama, Shinjuku-ku, Tokyo, 162-8636 Japan; Department of Bioscience and Biotechnology, Faculty of Bio-environmental Science, Kyoto Gakuen University, 1-1, Nanjo-Otani, Sogabe, Kameoka, Kyoto, 621-8555 Japan; Laboratory of Sports and Health Science, Kyoto Prefectural University of Medicine, 465 Kajii-cho, Kamigyo-ku, Kyoto, 602-0841 Japan

**Keywords:** Schoolchildren, Accelerometer, Step counts, Playing outdoors, Walking to school

## Abstract

**Background:**

The purpose of this study was to examine the change in accelerometer-derived daily physical activity (PA) and activity record-derived daily activities over a 10-year period in urban elementary schoolchildren in Japan.

**Methods:**

A total of 233 sixth-grade children (11–12 years old) in a same elementary school in Kyoto participated in the study (*n* = 125 and 108 in 1999 and 2009, respectively). The participant rate is 91.9 and 98.2 % in 1999 and 2009, respectively. The size and shape of the school district was not changed. The children were instructed to wear an accelerometer for 5 consecutive days of weekday and to keep minute-by-minute 24-h activity records with the assistance of their parents. The school-day scheduling was similar between 1999 and 2009 (29 and 27 sessions of 45-min class per week, respectively).

**Results:**

From 1999 to 2009, step counts considerably decreased (20,832 vs. 12,237 steps per day in boys and 16,087 vs. 10,748 steps per day in girls; *P* < 0.001) with concomitant significant decreases in total energy expenditure (*P* = 0.011), activity energy expenditure (*P* < 0.001), and physical activity level (*P* < 0.001). Time spent playing outdoors and walking to school were also significantly less in 2009 than 1999 (*P* < 0.001). Accelerometer-measured PA was significantly associated with the amount of time spent playing outdoors and walking to school.

**Conclusions:**

These results indicate that elementary schoolchildren in 2009 spend less time playing outdoors and walking to school, perform less PA, and take fewer steps than children of a decade ago.

## Background

There is an indirect evidence of a recent and considerable decrease in physical activity (PA) in schoolchildren. In the late 1990s, Rowlands et al. [1] reported that boys and girls in 8–10 years old from local primary school in Bangor, North Wales, averaged 16,035 ± 5999 and 12,729 ± 4026 steps per day (*P* < 0.05), respectively. In contrast, in 2007, McCormack et al. [2] found that a similar population of boys and girls in grades 5–7 attending public elementary schools in Perth, Western Australia, took 12,270 ± 3350 and 10,681 ± 2745 steps per day (*P* < 0.001), respectively. Likewise, in Japan, Kito et al. [3] reported in the early 1980s that boys and girls in 9–11 years old in Aich Prefecture took 18,812 and 14,048 steps per day, respectively, while Nemoto et al. [4] found that boys and girls in fourth-graders located in mountain area in Yamanashi Prefecture took 14,875 ± 4339 and 11,155 ± 2033 steps per day (*P* < 0.001) in 2009. Although not directly comparable, these results suggest that childhood PA might decrease over the past few decades; however, it must be noted that these separately conducted studies examined schoolchildren from different regions and populations and used different step counters, and thus they cannot be directly compared.

This is particularly true because previous studies have indicated that PA and step counts are regionally variable, even within the same country [5–7]. Itoi et al. [5] reported that the overweight and obesity prevalence in Japan was significantly higher in rural regions than in urban regions. The number of steps per day, activity energy expenditure (AEE), physical activity level (PAL), and duration of walking to school were significantly lower in rural children than in urban children. The PA and duration of the walk to school were significantly correlated with body mass index (BMI). Joens-Matre et al. [6] examined BMI and questionnaire-derived PA of fourth- to sixth-grade children in Iowa, USA. The prevalence of being overweight was higher, and PA was lower among rural children compared with urban children. Bassett et al. [7] found that Old Order Amish youth had high daily physical activity levels, as measured by a step counter and that obesity prevalence was quite low. Onywera et al. [8] reported that rural Kenyan children were more physically active than their urban counterparts, with a mean average steps per day (±SE) of 14,700 ± 521 and 11,717 ± 561 (*P* < 0.001) for rural and urban children, respectively. In addition, significantly different outputs are observed with different types of step counters or accelerometers. Studies are needed in which daily PA and step counts are measured using the same accelerometer in the same region over a long time period in order to reliably examine changes in the PA and lifestyle in schoolchildren.

Uechi et al. [9] examined the effect of playing outdoors during break between classes on daily PA in 9–12 years children and found that the children playing outdoors during breaks between classes had significantly higher total step counts (12,118 ± 3442 steps) compared with the children who did not play outdoors (9265 ± 2976 steps, *P* < 0.001). We previously compared urban and rural sixth-grade schoolchildren in Japan and found that rural children were less physically active because fewer of them walked to school owing to the prevalence of commuting by car [5]. Therefore, we hypothesized that the decline in PA in recent children relative to 1 decade ago was at least partly associated with decreases in time spent playing outdoors and walking to school.

The purpose of this study was to compare the daily PA levels of schoolchildren today with those of children 1 decade ago in a Japanese school located in Kyoto. We hypothesized (1) that the PA level would be lower at recent than 1 decade ago and (2) that lower daily PA levels would be associated with less time spent playing outdoors and walking to school.

## Methods

### Participants

A total of 233 sixth-grade children (11–12 years old) in an urban public elementary school in Kyoto, Japan, participated in the study. Sixty-two boys and 63 girls participated in 1999, and 56 boys and 52 girls participated in 2009. The data came from the same school in 1999 and 2009. In 1999, 136 sixth-grade children had been enrolled in the school, and we collected PA data from 91.9 % of children (*n* = 125). In 2009, 110 sixth-grade children had been enrolled in the school, and we collected PA data from 98.2 % of children (*n* = 108). Informed consent was obtained from all participating children, as well as their parents and teachers. The study was approved by the Ethics Committee of the Kyoto Prefectural University of Medicine (Reference number: RBMR-C-588).

Kyoto is one of the ten major cities in Japan; it has a population of over 1.47 million and a population density of 1780 people per km^2^. The size and shape of the school district were not changed between the two time points. Kyoto is one of the most historic cities in Japan, and this school locates one historical area. Because of preservation of historical areas, the rate of moving in the school district tends to lower than other urban cites. For socioeconomic status (SES), the number of vehicles owned (−11.3 %), appraised value of land (−21.8 %), and family income (−6.6 %) decreased in the 10 years in Kyoto city, which is similar to countrywide trend. Landscape between 1999 and 2009 in the school district was not much different. The school-day scheduling was also similar between 1999 and 2009 (29 and 27 sessions of 45-min class per week, respectively).

### Anthropometric characteristics

The age, grade, sex, and physical characteristics of the children were assessed. All measurements were conducted during the fall season. Body mass (kg) and height (cm) were measured to the nearest 0.1 kg and 0.1 cm, respectively, using a professional physician’s scale and stadiometer with the children standing barefoot and wearing one layer of light clothing. The BMI (kg · m^−2^) of each child was calculated as their body mass (kg) divided by their height squared (m^2^). Children were classified as overweight (OW) or obese (OB) according to the international definitions for childhood obesity that were developed in a workshop organized by the International Obesity Task Force (IOTF) [10]. IOTF cut off points for BMI for overweight and obesity by sex between 2 and 18 years was defined to pass through body mass index of 25 and 30 kg · m^−2^ at age 18, obtained by averaging data from Brazil, Great Britain, Hong Kong, the Netherlands, Singapore, and USA. For example, the corresponded BMI of OW is ≥20.55 in boys and ≥20.74 in girls, and that of OB is ≥25.10 in boys and ≥25.42 in girls for 11 years old.

### Accelerometer

A detailed description of the accelerometer used in this study has been provided elsewhere [5, 11–13]. A uniaxial pedometer/accelerometer (Kenz Lifecorder/Calorie counter; Kenz, Suzuken Co. Ltd., Nagoya, Japan) was continuously and rigidly attached to the waistband of each participating child during all waking hours for 5 consecutive days of weekdays, excluding time spent bathing or in water. The accelerometer is one of the most reliable pedometer/accelerometers for step counts [11, 14–16]. Japanese Industrial Standards Committee established a pedometer/accelerometer standard in 1993 (JIS S7200-1993; president of the committee, Dr. Yoshiro Hatano) and instructs to test pedometers with acceleration of 2.4 and 4.9 m/s^2^ induced by the oscillation generator with maximum acceptable error of ±3 %. The pedometer/accelerometers were checked and validated using the same Japanese Industrial Standards (JIS) method by the company before each survey in 1999 and 2009. The participants were asked to record all times and dates that they did not wear the accelerometer. The records and accelerometer data were checked, and the children were interviewed if a lack of compliance was suspected. The accelerometer data was accepted wearing of 10 h a day and 3 days above [17]. This accelerometer has been previously validated against both indirect calorimetry and the doubly labeled water method in adults and children [5, 11, 13, 18]. The reported margin of error in the number of steps recorded is less than 3 % [15, 16, 19]. Additionally, this accelerometer has superior step counting accuracy under both controlled [16, 20] and free-living conditions [15] in comparison with other instruments [21]. Although the total (TEE) and physical activity energy expenditure (AEE), and physical activity level (PAL) estimated by the accelerometer is highly correlated with the values obtained by the doubly labeled water method, the accelerometer underestimates TEE, AEE, and PAL [5, 13]. Thus, we adjusted these values using a correction factor validated in previous experiments [5]. Accelerometer data from eight children were excluded from analysis because of extremely low step counts due to long non-wear periods.

### 24-h activity records

Five-day minute-by-minute activity records were used to assess PA levels as described previously [5, 22]. Participants used a form specially designed to facilitate diary maintenance to record each activity minute-by-minute. They were instructed to record when their activity changed by drawing a line at the end of one activity under the corresponding time. A detailed demonstration and an example of a completed sample were given to the teachers, parents, and children before they began recording. We asked the teachers and parents to assist the children in completing their activity records. All activity records were checked, and any missing information was obtained. The participants were requested to continue with and record all of their regular daily activities. The activity record data from 17 children were excluded from analysis because records were missing, or the child had given up recording.

### Statistical analysis

Normal distribution was tested using a Kolmogorov-Smirnov test. Height, PAL, and duration of walking to school, watching TV, and sleeping, and bedtime showed normal distribution statistically. Two-way ANOVA was used with year (1999 or 2009) and sex (boy or girl) as between-subject factors for these variables. The other data of the variables showed no normal distribution. Mann-Whitney test was used to identify the effects of year and sex for these variables. Results are given as means, standard deviation (SD), median, and 5th and 95th percentiles of the variables. Differences in the distribution of weight status were examined using the chi-square test. The relationship between accelerometer-measured PA and duration of specific activities and between BMI and PA or duration of specific activities were examined using the Spearman rank correlation analysis. Significance was set at *P* < 0.05. Statistical analyses were conducted using SPSS statistics (Windows Version 22; SPSS Inc., Chicago, Illinois).

## Results

Table 1 shows the mean, SD, median, and 5th and 95th percentiles values for the variables with normal distribution. Table 2 shows the means, SD, median, and 5th and 95th percentiles values for the variables with non-normal distribution. The prevalence of obesity by IOTF is shown in Table 3.

**Table 1:** The variables showed normal distribution in Japanese schoolchildren in 1999 and 2009 (*n* = 233)

	1999									2009								*P* value of two-way ANOVA		
	Boys					Girls				Boys				Girls						
		Mean	(SD)	Median	(5th and 95th percentiles)	Mean	(SD)	Median	(5th and 95th percentiles)	Mean	(SD)	Median	(5th and 95th percentiles)	Mean	(SD)	Median	(5th and 95th percentiles)	Year	Sex	*Y* × *S*
	*n* = 62					*n* = 63				*n* = 56				*n* = 52						
Physical characteristics																				
Height	cm	145.8	(6.5)	145.1	(136.5–157.5)	147.1	(6.6)	148.0	(135.1–157.1)	147.8	(7.6)	148.4	(134.6–161.5)	150.1	(6.0)	149.8	(141.6–160.5)	*0.005*	*0.044*	0.615
Accelerometer output																				
PAL		1.92	(0.16)	1.89	(1.71–2.16)	1.85	(0.20)	1.83	(1.64–2.11)	1.72	(0.08)	1.72	(1.57–1.86)	1.73	(0.08)	1.73	(1.61–1.85)	*<0.001*	0.095	*0.046*
Time spent performing specific activities																				
Walking to school	min	41	(24)	38	(9–93)	46	(27)	40	(5–98)	35	(20)	34	(5–70)	39	(22)	40	(8–73)	*0.043*	0.202	0.954
Watching TV	min	108	(61)	105	(13–229)	98	(61)	96	(3–211)	90	(57)	84	(1–198)	111	(84)	92	(8–254)	0.775	0.549	0.088
Sleeping	min	515	(48)	523	(427–583)	502	(42)	510	(427–570)	500	(41)	503	(421–568)	490	(50)	498	(392–574)	*0.029*	0.069	0.790
Bedtime	h:m	22:30	(51')	22:20	(21:00–00:01)	22:42	(44')	22:43	(21:28–00:00)	22:36	(42')	22:28	(21:37-23:36)	22:47	(51')	22:48	(21:18–00:17)	0.435	0.070	0.985

*PAL* physical activity level; Significance was set at P < 0.05

**Table 2:** The variables showed no normal distribution in Japanese schoolchildren in 1999 and 2009 (*n* = 233)

		1999								2009								Mann-Whitney Test	
		Boys				Girls				Boys				Girls					
		Mean	(SD)	Median	(5th and 95th percentiles)	Mean	(SD)	Median	(5th and 95th percentiles)	Mean	(SD)	Median	(5th and 95th percentiles)	Mean	(SD)	Median	(5th and 95th percentiles)	Year	Sex
		*n* = 62				*n* = 63				*n* = 56				*n* = 52					
Physical characteristics																			
Weight	kg	37.1	(6.3)	35.9	(29.1–51.5)	38.5	(6.7)	38.2	(28.8–53.0)	39.6	(8.3)	39.5	(27.5–57.4)	41.7	(7.4)	40.9	(31.4–57.3)	*0.003*	*0.042*
BMI	kg · m^−2^	17.4	(2.3)	16.7	(14.7–22.0)	17.7	(2.3)	17.4	(14.2–22.4)	18.0	(2.6)	17.8	(14.0–23.9)	18.5	(2.5)	18.3	(15.2–23.8)	*0.024*	0.125
Accelerometer output																			
Step count	step	20,832	(4694)	20,748	(14,277–30,806)	16,087	(4840)	15,720	(8336–25,330)	12,237	(2934)	12,014	(7492–18,324)	10,748	(2668)	11,226	(6278–14,824)	*<0.001*	*<0.001*
TEE	kcal	2512	(381)	2434	(1977–3258)	2318	(398)	2268	(1652–3065)	2303	(383)	2337	(1677–3036)	2245	(285)	2234	(1765–2790)	*0.011*	*0.012*
AEE	kcal	805	(247)	744	(463–1309)	626	(213)	592	(332–1056)	408	(118)	408	(214–648)	367	(100)	391	(202–537)	*<0.001*	*0.002*
Time spent performing specific activities																			
Playing outdoor	min	46	(36)	42	(0–114)	31	(29)	25	(0–81)	31	(33)	17	(0–109)	13	(14)	11	(0–47)	*<0.001*	*0.002*
Club activity	min	26	(43)	16	(0–86)	28	(36)	13	(0–91)	28	(22)	25	(0–85)	15	(17)	16	(0–59)	0.473	0.086
Video game	min	29	(40)	13	(0–123)	8	(19)	0	(0–45)	29	(34)	22	(0–104)	24	(55)	6	(0–93)	*0.011*	*<0.001*
Hobby	min	13	(19)	5	(0–52)	17	(18)	13	(0–60)	9	(16)	2	(0–39)	20	(24)	12	(0–77)	0.406	*0.001*
Studying	min	62	(75)	39	(3–276)	66	(79)	36	(0–288)	46	(37)	38	(0–121)	46	(34)	36	(7–125)	0.606	0.884
Awake time	h:m	7:02	(20')	7:00	(6:30–7:40)	7:04	(27')	7:09	(5:59–7:44)	6:56	(30')	7:01	(5:54–7:42)	6:58	(31')	7:01	(6:00–7:37)	0.195	0.253

*BMI* body mass index, *TEE* total energy expenditure, *AEE* activity energy expenditure; Significance was set at P < 0.05

**Table 3:** The prevalence of obesity by IOTF criteria obesity

		1999		2009	
		Boys	Girls	Boys	Girls
		*n* = 62	*n* = 63	*n* = 56	*n* = 52
Overweight	%	4.8	6.3	8.9	11.5
Obesity	%	1.6	1.6	1.8	1.9

*IOTF* International Obesity Task Force

### Physical characteristics

Height (*F*_(229,1)_ = 7.896, *P =* 0.005), weight (*U* = 8295, *P =* 0.003), and BMI (*U* = 7905, *P* = 0.024) were significantly higher in 2009 than in 1999. Slightly, more children were classified as overweight or obese in 2009 compared with 1999, but this difference did not reach statistical significance (*P* > 0.05).

### Accelerometers output

In 2009, the children took fewer steps (*U* = 1473, *P* < 0.001) and had lower TEE (*U* = 5009, *P* = 0.011), AEE (*U* = 1100, *P* < 0.001), and PAL (*F*_(221,1)_ = 69.4, *P* < 0.001) than the children in 1999. Girls had lower step counts (*U* = 4320, *P* < 0.001), TEE (*U* = 5102, *P* = 0.012), AEE (*U* = 4834, *P* = 0.002), and PAL (*F*_(221,1)_ = 2.818, *P* = 0.095) than boys. Significant year and sex interactions were observed in PAL (*F*_(221,1)_ = 4.025, *P* = 0.046), and the secular decline is greater for boys, compared to girls.

### Time spent performing specific activities

In 2009, the children spent less time playing outdoors (*U* = 3930, *P* < 0.001) and walking to school (*F*_(191,1)_ = 4.159, *P* = 0.043) than in 1999. The duration of sleeping (*F*_(212,1)_ = 4.828, *P =* 0.029) was also significantly less in 2009 than in 1999. The duration of video games (*U* = 6926, *P =* 0.011) was significantly longer in 2009 than in 1999. No significant interactions between year and sex were observed for any of the variables (Tables 1, 2, and 3).

Time spent playing outdoors was significantly correlated with accelerometer-measured PA in 1999 (for step count, *ρ* = 0.300, *P* = 0.001; TEE, *ρ* = 0.243, *P* = 0.010; AEE, *ρ* = 0.295, *P* = 0.002; PAL, *ρ* = 0.432, *P* < 0.001). Time spent playing outdoors was significantly correlated with accelerometer-measured PA also in 2009 (for step count, *ρ* = 0.413, *P* < 0.001; AEE, *ρ* = 0.360, *P* < 0.001; PAL, *ρ* = 0.309, *P* = 0.002). Time spent playing outdoors was significantly correlated with accelerometer-measured PA in boy (for step count, *ρ* = 0.293, *P* = 0.003; TEE, *ρ* = 0.271, *P* = 0.006; AEE, *ρ* = 0.346, *P* < 0.001; PAL, *ρ* = 0.410, *P* < 0.001). Time spent playing outdoors was significantly correlated with accelerometer-measured PA in girl as well (for step count, *ρ* = 0.427, *P* < 0.001; AEE, *ρ* = 0.424, *P* < 0.001; PAL, *ρ* = 0.438, *P* < 0.001). Time spent walking to school was not significantly correlated with accelerometer-measured PA in 1999, but was significantly correlated with accelerometer-measured PA in 2009 (for step count, *ρ* = 0.451, *P* < 0.001; AEE, *ρ* = 0.290, *P* = 0.005; PAL, *ρ* = 0.363, *P* < 0.001). Time spent walking to school was significantly correlated with accelerometer-measured PA in boy (for step count, *ρ* = 0.277, *P* = 0.007). Time spent walking to school was significantly correlated with accelerometer-measured PA in girl as well (for step count, *ρ* = 0.309, *P* = 0.002; PAL, *ρ* = 0.262, *P* = 0.011). BMI was significantly and negatively correlated with step counts (*ρ* =−0.281, *P* < 0.001), duration of sleeping (*ρ* =−0.162, *P* = 0.017), and positively correlated with bedtime (*ρ* = 0.167, *P* = 0.014).

## Discussion

Our results clearly show that children in a Japanese elementary school had lower step counts (*P* < 0.001), TEE (*P =* 0.011), AEE, (*P* < 0.001), and PAL (*P* < 0.001) in 2009 than 1 decade earlier and spent less time playing outdoors (*P* < 0.001) and walking to school (*P =* 0.043). Although the prevalence of overweight and obesity was not significantly higher in 2009 than in 1999, the average BMI was significantly higher in 2009 (*P =* 0.024).

In 2004, Tudor-Locke et al. [23] recommended 15,000 and 12,000 steps per day for boys and girls, respectively. In the present study, 90.3 % of boys and 82.5 % of girls in 1999 reached these recommended step counts. In 2009, the percentages of boys and girls who achieved the recommended step counts were only 15.7 % and 40.8 %, respectively—significantly lower than the 1999 values (*P* < 0.001). The step counts in 2009 in the present study are quite similar to previous results by McCormack et al. [2] in 2007 and Nemoto et al. [4] in 2009 examination. In contrast, the step counts in 1999 in the present study were 20,832 ± 4694 and 16,087 ± 4840 for boys and girls, respectively. These values are relatively higher than previous reports by Rowlands et al. [1] in late 1990s and Kito et al. [3] in early 1980s. A possible reason for this is that the elementary school examined in the present study is located at the boundary of an urban area and serves a geographically larger school district than typical Japanese urban schools. The children had been instructed to walk to school even their house is over 2 km apart from school. However, the increased perception in parents for risks of accidents during walk to school only children, some parents drive their children partway recently. The decrease in the amount of time spent walking to school over the 10-year study period was mostly due to an increase in the proportion of children being driven partway by parents in the morning. Previous studies have also found a decrease in active transportation to school in Switzerland [24] and the USA [25]. Reasons that have been suggested for this trend include increased distances to school and, where distances have not increased, reduced availability of bikes and increased numbers of cars per household. Additionally, increased awareness of, and anxiety about, motor vehicle accidents that may occur while walking to school in Japan may also play a role in the tendency of parents to drive their children to school.

Cooper et al. [26] reported that children who walked to school had 43 % higher mean accelerometer counts per minute before school than those traveling by car. Loucaides and Jago [27] reported that children who walked to school had higher step counts during the before- and after-school period, and the whole day, in comparison to children who used motorized transport. It has been reported that rural children in Japan perform less PA, and especially spend less time walking to school, than urban children [5]. Thus, walking to school is an important factor in determining daily physical activity, and increasing the number of children who walk to school could be a major strategy for promoting childhood health.

Tudor-Locke et al. [28] reported in 2006 that boys took significantly more total steps per day than girls, including more steps during recess, lunchtime, and after-school. Girls took more steps than boys before school, but boys and girls took the same number of steps during structured physical education classes. Lunchtime PA represented the most important source of daily PA (15–16 %) obtained during school hours for both boys and girls, whereas recess accounted for 8–9 % and physical education class accounted for 8–11 % of the students’ total steps per day. However, almost half of the daily steps taken by schoolchildren are attributable to after-school activities. Nemoto et al. [4] reported that boys had higher step counts and PA than girls, and that this was associated with the amount of time spent playing outdoors. In the present study, we found that boys took significantly more total steps per day, and had greater TEE and PAEE, than girls. Our findings indicate that in 2009, children spent less time playing outdoors and walking to school than did the children in 1999, and the time spent playing outdoors was significantly correlated with the PA measured by the accelerometer; these findings are similar to those of previous studies. Haug et al. [29] reported that students with access to more outdoor facilities were three times more likely to be active during recess than students with access to fewer facilities. Thus, physical environment may be a factor that determines the PA of schoolchildren, and altering the environment may be one way to increase PA on a population level.

It is important to note that body composition, such as percent body fat or skeletal and muscle development, should be examined not only BMI. Scheffler, Rietsch, and their colleagues examined secular trends of height, BMI, and external skeletal robustness (Frame-Index or pelvic breadth) and the relationship between PA and BMI, percent body fat, and Frame-Index [30–34]. That means that two variables correlate with BMI—a fat content and a skeletal part. This indicates that BMI is not merely high due to a high body fat content but rather due to high skeletal part. Low physical activity can cause the reduction of external skeletal robustness, the increase of BMI and body fat. They demonstrate an association between external skeletal robustness and PA, which is not captured in BMI measurements. We found significantly but weak correlations between BMI and step counts, duration of sleeping, and bedtime. We did not examine any body composition in this study, and this is a limitation of this study.

Sigmundova et al. [35] reported that prevalence of overweight and obesity in Czech adolescents increased from 5.5 % in 1998–2000 to 10.4 % in 2008–2010, step count in weekdays, but not in weekends, significantly decreased, and self-reported walking and aerobic exercise duration decreased between those two cohorts. Sigmund et al. [36] reported similar trend of increased obesity prevalence and decreased moderate-to-vigorous intensity PA between 2002 and 2014 in Czech adolescents. Booth et al. [37] reviewed previous researches and found mixed results, and inconsistent magnitudes of change were identified when exploring trends in different contexts of physical activity for children and adolescents over the last few decades. Taken overall, organized sport participation was increased: consistent declines in active transport were found in all of seven studies investigating active transport. Because few studies investigated the secular trend of PA or step count using objective methods, they stated that need more evidence to conclude. Our finding about the secular trends of decreasing objectively measured step counts and PA in Japanese children supports the importance of PA to their healthy development. As Booth et al. [37] found from the review, consistent declines in active transport highlight this context as a suitable intervention target.

The information technology environment has changed markedly in recent years, and these changes have transformed children’s play in daily life. Nelson et al. [38] reported that secular trends further indicated dramatic increases in mid-adolescent weekly computer use from 1999 to 2004; from 8.8 to 11.1 h, and 10.4 to 15.2 h in girls and boys, respectively. We could not examine the effect of computer or smartphone use on children’s PA in the present study, and further research is needed in this area. Another limitation of the present study was that all of the participants were from just one elementary school, and therefore, the results may not be applicable to all populations in Japan. However, the decrease in step counts that we observed is similar to that reported in adults in a recent government report [39]. In addition, we did not examine activity in weekends, organized sport participation, as well as body composition, maturational status, and height and weight growth velocity [40]. As another potential confounders, we did not examine the effect of climate and weather, or SES and education status of parents on children’s PA. Further, large-scale researches are needed to examine these issues in more depth using random sampling.

## Conclusions

The step counts, TEE, AEE, and PAL of Japanese urban schoolchildren were significantly lower in 2009 than 1 decade earlier in the same region. Significantly, less time was spent playing outdoors and walking to school in 2009 than was spent 1 decade ago. The average BMI was significantly higher in 2009 compared with 1999, although the prevalence of overweight and obesity was not significantly different. Physical activity was significantly correlated with time spent playing outdoors and walking to school. Modern children have lower physical activity levels than the children of a decade ago, and this may be at least partly a result of less time being spent playing outdoors or active transport to school.

## Abbreviations

AEE: activity energy expenditure
BMI: body mass index
JIS: Japanese Industrial Standards
PA: physical activity
PAL: physical activity level
TEE: total energy expenditure
